# Kampo formula hochu-ekki-to (Bu-Zhong-Yi-Qi-Tang, TJ-41) ameliorates muscle atrophy by modulating atrogenes and AMPK in vivo and in vitro

**DOI:** 10.1186/s12906-022-03812-w

**Published:** 2022-12-28

**Authors:** Mitsutaka Yakabe, Tatsuya Hosoi, Hiroko Sasakawa, Masahiro Akishita, Sumito Ogawa

**Affiliations:** Department of Geriatric Medicine, Graduate School of Medicine, The University of Tokyo, 7- 3-1, Hongo, Bunkyo-ku, 113-8655 Tokyo, Japan

**Keywords:** TJ-41, Tail-suspension, atrogin-1, AMPK, Herbal medicine

## Abstract

**Background:**

Muscle disuse results in loss of skeletal muscle mass and function. Hochu-ekki-to (TJ-41; Bu-Zhong-Yi-Qi-Tang in Chinese) is an herbal medicinal formulation used to treat patients with frailty, fatigue and appetite loss. It has been suggested that two atrogenes, atrogin-1 and muscle Ring finger 1 (MuRF1), are ubiquitin ligases involved in disuse-induced muscle atrophy and that 5’ adenosine monophosphate-activated protein kinase (AMPK) is involved in skeletal muscle metabolism. Effects of TJ-41 on disuse-induced muscle atrophy are unclear.

**Methods:**

We subjected differentiated C2C12 myotubes to serum starvation, then examined the effects of TJ-41 on atrogenes expression, AMPK activity and the morphology of the myotubes. Male C57BL/6J mice were subjected to tail-suspension to induce hindlimb atrophy. We administered TJ-41 by gavage to the control group and the tail-suspended group, then examined the effects of TJ-41 on atrogene expression, AMPK activity, and the muscle weight.

**Results:**

Serum starvation induced the expression of atrogin-1 and MuRF1 in C2C12 myotubes, and TJ-41 significantly downregulated the expression of atrogin-1. Tail-suspension of the mice induced the expression of atrogin-1 and MuRF1 in skeletal muscle as well as its muscle atrophy, whereas TJ-41 treatment significantly downregulated the expression of atrogin-1 and ameliorated the loss of the muscle weight. In addition, TJ-41 also activated AMPK and inactivated Akt and mTOR in skeletal muscle in vivo.

**Conclusion:**

TJ-41 inhibited atrogenes in an Akt-independent manner as well as activating AMPK in skeletal muscles in vivo, further implying the therapeutic potential of TJ-41 against disuse-induced muscle atrophy and other atrogenes-dependent atrophic conditions.

**Supplementary Information:**

The online version contains supplementary material available at 10.1186/s12906-022-03812-w.

## Background

Muscle disuse is one cause of skeletal muscle mass and function loss. So far, no treatment methods have been established for disuse-induced muscle atrophy.

Atrogenes atrogin-1 and MuRF1 are ubiquitin ligases involved in muscle atrophy, and skeletal muscles of mice deficient in either of these genes are resistant to muscle atrophy [1]. These atrogenes are upregulated during disuse-induced atrophy conditions, such as tail-suspension, denervation, and immobilization [2]. These genes are reported to be regulated by the phosphoinositide 3-kinase (PI3K)/Akt/ mammalian target of rapamycin (mTOR) pathway and forkhead box protein O (FoxO) transcriptional factors [3–5].

In addition, 5’ adenosine monophosphate-activated protein kinase (AMPK) has two kinds of effects on metabolism: the inhibition of anabolism to minimize ATP consumption and the stimulation of catabolism to stimulate ATP production [6], and it is involved in skeletal muscle metabolic control [7]. AMPK is activated by repeated muscle contraction and exercise.

Hochu-ekki-to (TJ-41; Bu-Zhong-Yi-Qi-Tang in Chinese) is an herbal medicinal formulation that consists of ten types of single herbs (shown in Table 1) [8]. TJ-41 is mainly used to treat elderly patients that experience frailty, fatigue, and loss of appetite clinically [9, 10].

**Table 1:** The ingredients of TJ-41

Herbs	Weight
Ginseng Radix (roots of *Panax ginseng*)	4 g
Astragali Radix (roots of *Astragalus membranaceus*)	4 g
Atractylodis lanceae Rhizoma (rhizomes of *Atractylodes lancea*)	4 g
Bupleuri Radix (roots of *Bupleurum falcatum*)	2 g
Angelicae Radix (roots of *Angelica acutiloba*)	3 g
Cimicifugae Rhizoma (rhizomes of *Cimicifuga simplex*)	1 g
Aurantii Bobilis Pericarpium (pericarps of ripe fruits of *Citrus unshiu*)	2 g
Zingiberis Rhizoma (rhizomes of *Zingiber officinale*)	0.5 g
Zizyphi Fructus (fruits of *Zizyphus jujube*)	2 g
Glycyrrhizae Radix (roots of *Glycyrrhiza uralensis*)	1.5 g

Some studies have examined about the effects of herbal medicinal formulations on skeletal muscles. For example, Go-sha-jinki-gan ameliorates the loss of muscle mass in senescence-accelerated mice [11]. Other herbal medicinal formulations, such as Rikkunshito and Qing-Shu-Yi-Qi-Tang ameliorated reductions in body weight and muscle weight in mice with cancer and cachexia [12]. A combination of Qing-Shu-Yi-Qi-Tang and Scutellaria baicalensis decreased the expressions of nuclear factor-kappa B (NF-κB) and MuRF1 in the gastrocnemius muscle of tumor-bearing mice [13].

It is known that TJ-41 has immunomodulatory and anti-inflammatory effects in vitro and in vivo studies [14–17]. TJ-41 ameliorated ultraviolet-B-induced oxidative stress and skin dysfunction in hairless mice [18]. TJ-41 exerted anti-asthmatic effects by relieving airway hyper-responsiveness in a mouse model of allergic asthma [19]. In clinical studies, the administration of TJ-41 significantly decreases serum TNF-α and IL-6 levels in patients of chronic obstructive pulmonary disease [20, 21]. However, few studies have investigated the effects of TJ-41 on skeletal muscle. A study showed that TJ-41 downregulated nuclear receptor corepressor 1 (NCoR1) expression, and induced muscle differentiation and metabolism by regulating NCoR1associated gene expression in weightlessness-induced muscle atrophy [22]. TJ-41 also inhibited inflammation and oxidative stress in the gastrocnemius muscles of amyotrophic lateral sclerosis model mice, and extended the survival of the mice [23]. TJ-41 ameliorated reductions in body weight and skeletal muscle weight in cachexic tumor-bearing mice by inhibiting the production of IL-6 by host cells such as macrophages [24]. Atrogenes are implicated in the process of muscle atrophy in models of cachexia [25]. However, no study has examined the effects of TJ-41 on atrogenes and AMPK activation.

In the study, we hypothesized that TJ-41 might possess therapeutic potential against disuse-induced muscle atrophy. We examined in vivo and in vitro effects of TJ-41 on atrogenes and AMPK regulation as well as related muscle atrophy, thereby focusing on its therapeutic basis against muscle atrophy. For in vivo analysis, tail-suspended mice were adopted as a disuse-induced muscle atrophy model.

## Methods

### Materials

Dulbecco’s modified essential medium (DMEM) was purchased from Nikken Bio Med Lab (Kyoto, Japan), fetal bovine serum (FBS) from Biowest (Nuaillé, France), horse serum and penicillin-streptomycin from Thermo Fisher Scientific (Waltham, MA, USA). Human insulin and the primary antibodies targeting β-actin and β-tubulin were purchased from Sigma-Aldrich Inc. (St. Louis, MO, USA). The primary antibodies targeting p-Akt, Akt, p-p70, p70, p-mTOR, mTOR, p-FoxO1, p-AMPK, and AMPK were purchased from Cell Signaling (Beverly, MA, USA). The secondary antibodies, anti-rabbit IgG, and anti-mouse IgG were purchased from GE Healthcare (Bucks, UK). The C2C12 cells were obtained from the American Type Culture Collection (Manassas, VA, USA).

The dried extract preparation of TJ-41 (lot no. 2,140,041,010) was kindly provided by Tsumura & Co. (Tokyo, Japan). For in vitro experiments, one gram of TJ-41 was sonicated in 10 mL of dimethyl sulfoxide (DMSO), centrifuged, and the supernatant was harvested and diluted with DMSO to the appropriate concentration. For in vivo experiments, TJ-41 was mixed with water, sonicated, and diluted with water to a specific concentration.

### *In vitro* experiment

The C2C12 cells, a murine myoblast cell line, were cultured in DMEM supplemented with 10% FBS, 100 U/mL penicillin, and 100 µg/mL streptomycin at 37 °C in a humidified atmosphere (5% CO_2_ and 95% air).

Myoblasts fuse into multinucleated fibers called myotubes, and matured myotubes form muscle fibers [26]. To examine the effects of TJ-41, therefore, we differentiated C2C12 myoblast into myotubes in all the in vitro experiments. When cell confluency reached 80–90%, the medium was replaced with differentiation medium consisting of DMEM supplemented with 2% horse serum, 100 U/mL penicillin, and 100 µg/mL streptomycin. The differentiation medium (DM) was changed every 2 days. Four days later, the C2C12 cells differentiated into myotubes and were then used for the experiments.

Serum-free medium (FM) consisted of DMEM supplemented with 2% 100 U/mL penicillin and 100 µg/mL streptomycin but without serum. Atrophy was induced by replacing the DM of the differentiated C2C12 myotubes with the FM and culturing them for another 24 h (serum starvation). The control C2C12 myotubes were incubated for another 24 h after the replenishment of the DM. Then RNA was collected from the control group and serum-starved group using a Qiashredder and RNeasy Mini Kit (Qiagen, Hilden, Germany) and subjected to RNA analysis.

Previous studies have shown that TJ-41 concentration-dependently reduced IL-6 production from macrophages and restored autophagy in HEK293 cells in the concentration range of 10–500 µg/mL [24, 27]. TJ-41 concentration in the present study was determined based on these studies. The presence of 0.1% DMSO (v/v) in the medium affected the viability of C2C12 only slightly [28], and the concentration has been commonly adopted in the C2C12 experiments [29]. Therefore, we set the final DMSO concentration to 0.1% (v/v) in all the experiments.

To examine the effects of TJ-41 on atrogene expression, the DM of the differentiated C2C12 myotubes was changed to FM containing vehicle (0.1% DMSO v/v) or TJ-41 (final concentrations of 1, 10, or 100 µg/mL in medium, containing 0.1% DMSO v/v). The DM of the control group was replaced with DM containing DMSO (vehicle, 0.1% DMSO v/v). After 24 h incubation, RNA was collected from these groups using a Qiashredder and RNeasy Mini Kit (Qiagen) and subjected to RNA analysis.

To examine the effects of TJ-41 on the signaling pathway related to atrogenes, the DM of the C2C12 myotubes was replaced with the DM containing vehicle (0.1% DMSO v/v) or TJ-41 (final concentrations of 1, 10, or 100 µg/mL in medium, containing 0.1% DMSO v/v). After additional 24 h incubation, protein samples were collected and subjected to Western blot.

In all these experiments, three biological replicates and two technical replicates were performed.

### Animal experiments

Male C57BL/6J mice were purchased from Nippon CLEA (Tokyo, Japan). They were housed individually in similarly-designed cages and were maintained in a controlled environment (temperature, 24 ± 1 °C; humidity, 55 ± 5%) with a 12 h:12 h light:dark cycle. The mice were provided ad libitum access to standard chow and water. They were acclimated for 2 weeks in the aforementioned conditions and were used for the experiments at 11 weeks of age.

TJ-41 was mixed with water, sonicated, and orally administered by gavage. Control mice were administered water as a vehicle. The low-dose and high-dose groups received 0.3 and 1.0 g TJ-41 per kg body weight, respectively. Gavage feeding was performed daily during the experiments.

To examine the effects of TJ-41 on skeletal muscles of the wildtype mice, mice were administered water (vehicle), low-dose of TJ-41, or high-dose of TJ-41 by gavage daily for 21 days (*n* = 4 in each group, total number is 12). Then muscle samples were collected and subjected to Western blot.

The mice were subjected to tail-suspension, which induced hindlimb muscle atrophy. To achieve this, the entire tail of a mouse was covered with medical adhesive tape. The distal end of the tape was attached to a paperclip, which was then attached to a swivel on a cross-bar. The cross-bar was positioned approximately 15 cm above the cage floor. Using this device, the mouse was suspended such that the hindlimbs did not touch the floor, but the forelimbs were free to move. During this time, the mouse had ad libitum access to food and water. To examine the effects of tail-suspension on atrogene expression and muscle weight,

To examine the effects of tail-suspension on the expression of atrogin-1 and MuRF1 in the muscles, mice were subjected to 24 h of tail-suspension, and RNA was collected from the gastrocnemius muscles. To examine the effects of tail-suspension on muscle weight, mice were subjected to 14 days of tail-suspension and the gastrocnemius muscles were weighed. These data were compared with that of the control group (*n* = 4 in each group, total number is 12).

To examine changes in atrogenes expression in response to TJ-41, mice were divided into six groups: (1) non-suspended, vehicle (water), (2) non-suspended, low-dose TJ-41, (3) non-suspended, high-dose TJ-41, (4) suspended, vehicle, (5) suspended, low-dose TJ-41, (6) suspended, high-dose TJ-41 (*n* value is 10, 10, 10, 9, 10, 9 in each group, respectively, and total number is 58). The mice were subjected to 7 days of everyday treatment with vehicle (water) or TJ-41, followed by 24 h of non-suspended state or tail-suspension, then sacrificed. They were sacrificed 24 h after the last gavage. RNA was collected from the gastrocnemius muscles.

To examine the effects of TJ-41 on tail-suspension-induced muscle atrophy, another set of mice were divided into six groups: (1) non-suspended, vehicle, (2) non-suspended, low-dose TJ-41, (3) non-suspended, high-dose TJ-41, (4) suspended, vehicle, (5) suspended, low-dose TJ-41, (6) suspended, high-dose TJ-41 *n* value is 9, 10, 10, 9, 10, 8 in each group, respectively, and total number is 56). Mice were subjected to pretreatment for 7 days, then non-suspended state or tail-suspension for 14 days. During the time course, the mice were administered vehicle or TJ-41 every day. Then they were sacrificed and the gastrocnemius muscles were collected and weighed. They were sacrificed 24 h after the last gavage.

In all the animal experiments, the mouse was euthanized using carbon dioxide. Mice which had accidentally escaped from the suspension before the end of the experiments were excluded. In animal experiments, including and excluding criteria were not set a priori.

### RNA analysis

RNA was extracted from C2C12 myotubes, using a Qiashredder and RNeasy Mini Kit (Qiagen). Harvested gastrocnemius muscles were immersed in RNAlater (Qiagen) to stabilize RNA in tissues. RNA extraction was performed using an RNeasy Fibrous Tissue Mini Kit (Qiagen). DNase I treatment was performed using an RNase-Free DNase Set (Qiagen).

The cDNA was synthesized from the RNA samples using a ReverTra Ace qPCR RT Kit (Toyobo, Osaka, Japan) and used according to the manufacturer’s instructions. The cDNA was mixed with SYBR Green Master Mix (Applied Biosystems, CA, USA) and specific primers for each gene, and then subjected to quantitative real-time PCR using a StepOnePlus Real-Time PCR System (Applied Biosystems). The cycle threshold for each gene was normalized to that of glyceraldehyde-3-phosphate dehydrogenase (GAPDH), which was used as the internal control. The primer sequences were:

GAPDH: 5’-AGGTCGGTGTGAACGGATTTG-3’ (forward) and 5’-TGTAGACCAGTAGTTGAGGTCA-3’ (reverse);

MuRF1: 5’-TGCCTGGAGATGTTTACCAAGC-3’ (forward) and 5’-AAACGACCTCCAGACATGGACA-3’ (reverse);

atrogin-1: 5’-AAGGCTGTTGGAGCTGATAG CA-3’ (forward) and 5’-CACCCACATGTTAATGTTGCCC-3’ (reverse).

### Western blot

The C2C12 myotubes were lysed in radioimmunoprecipitation assay buffer combined with a protease inhibitor cocktail (cOmplete Mini, Roche Applied Science, Penzberg, Germany) and a phosphatase inhibitor cocktail (PhosSTOP, Roche Applied Science). Harvested muscle tissue was immersed in ice-cold T-PER Tissue Protein Extraction Reagent (Thermo Fisher Scientific) with cOmplete Mini and PhosSTOP, and pulverized using a CellDestroyer (Bio Medical Science Inc, Tokyo, Japan). The proteins in these lysates were separated using SDS-PAGE and were electro-transferred onto a polyvinylidene difluoride membrane. The membranes were blocked using Blocking One (Nacalai Tesque Inc, Kyoto, Japan) and probed using appropriate primary and secondary antibodies. After immunoblotting, the proteins were visualized using an ECL detection system (GE Healthcare) or LumiGLO Reserve Chemiluminescent Substrate (SeraCare Life Sciences, Inc. MA, USA). The chemiluminescence images were scanned using a LuminoGraph I (Atto Corp, Tokyo, Japan). Blots were quantified using Image J software 1.44.

### Cell morphology

To examine the effects of TJ-41 on serum-starved C2C12 myotube width, the DM of the differentiated C2C12 myotubes was changed to FM containing vehicle (0.1% DMSO v/v) or TJ-41 (final concentrations of 10 or 100 µg/mL in medium, containing 0.1% DMSO v/v). The DM of the control was replaced with DM containing DMSO (vehicle, 0.1% DMSO v/v). After 24 h incubation, the C2C12 myotubes were fixed using 4% paraformaldehyde in PBS solution (Wako, Osaka, Japan). To visualize cell morphology, the myotubes were treated with Actin Green 488 ReadyProbes reagent (Thermo Fisher Scientific). The images were scanned using a BZ-X810 fluorescence microscope (Keyence, Tokyo, Japan). Myotube width was determined using ImageJ (National Institutes of Health, Bethesda, MD, USA). Three independent biological replicates were performed, and three different fields were randomly selected from each experiment. Up to 20 representative fibers were selected from each field, and the width of each fiber was measured.

### Statistical analysis

The results were expressed as mean ± standard error of the mean values. Between-group comparisons were performed using Student’s t-tests. Multiple-group comparisons were performed using ANOVA, then post-hoc comparison was performed using the Tukey-Kramer method. Data analysis was conducted using R software. A value of *p* < 0.05 was considered significant.

## Results

### TJ-41 downregulated atrogin-1 in C2C12 myotubes

**Fig. 1:**
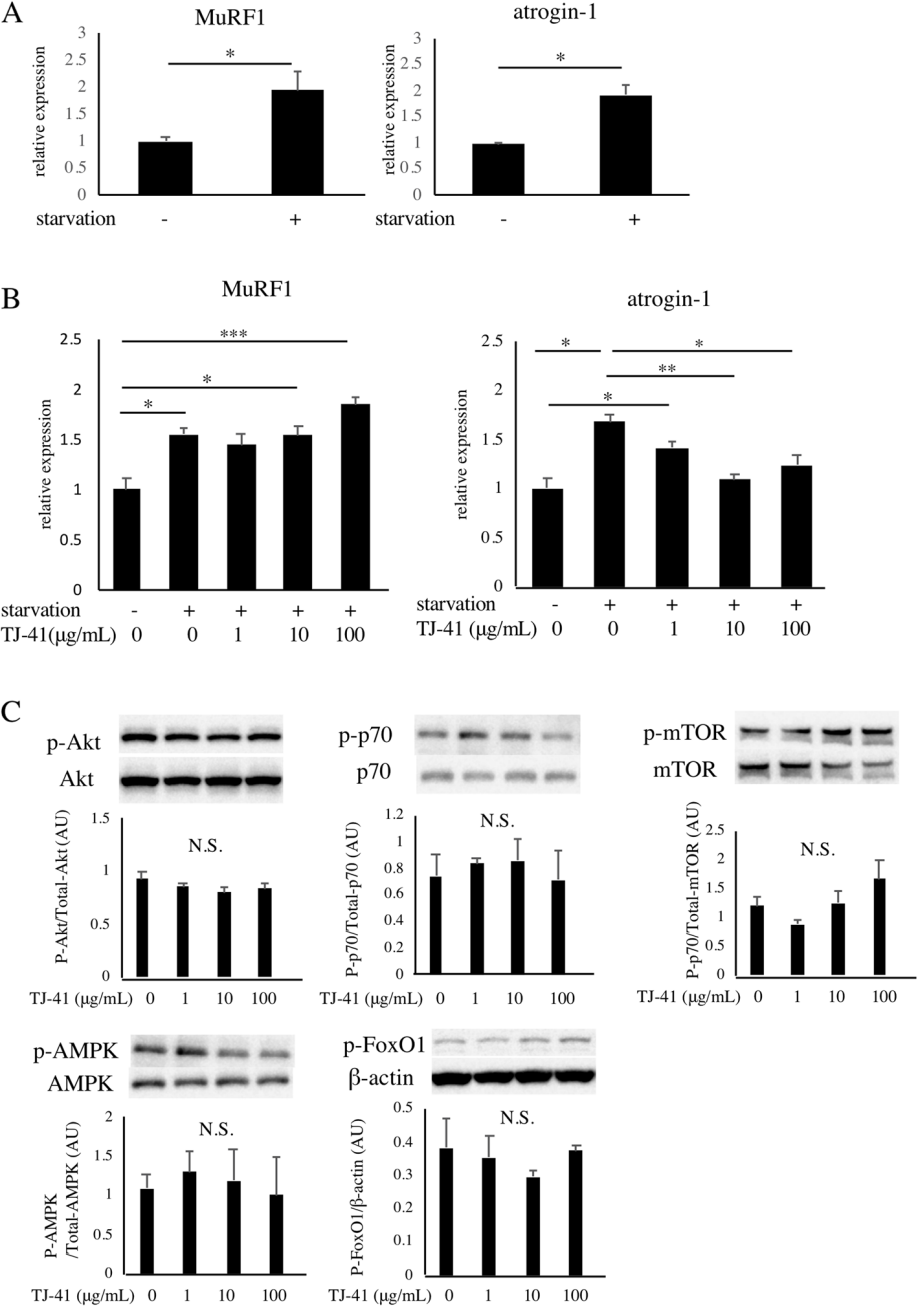
Effects of TJ-41 on C2C12 myotubes. **A** Expression of atrogin-1 and MuRF1 after 24 h of serum deprivation (*n* = 3). **B **Effects of TJ-41 extraction on expression of atrogin-1 and MuRF1. Concentrations of the extract are 1, 10 or 100 µg/mL (*n* = 3). Significant differences were detected by the post-hoc Tukey-Kramer test. **B** Phosphorylation of Akt, p70, mTOR, FoxO1, and AMPK in TJ-41-treated myotubes (western blot). These images were cropped from the original gels and blots. *p < 0.05, **p < 0.01, ***p < 0.001. NS indicates “not significant”

First, we examined the effects of TJ-41 on atrogenes in vitro. We adopted serum-starved C2C12 myotubes, an in vitro muscle atrophy model [30, 31]. C2C12 myotubes that were cultured in the FM for 24 h displayed significantly upregulated MuRF1 and atrogin-1 (*p* < 0.05) (Fig. 1A). We also cultured C2C12 myotubes in the FM with the vehicle or TJ-41 extract for 24 h. TJ-41 repressed atrogin-1, but not MuRF1 (Fig. 1B). Post-hoc Tukey-Kramer test showed that 10 and 100 µg/mL of TJ-41 significantly downregulated atrogin-1 (*p* < 0.01). We also assessed Akt, p70, mTOR, FoxO1, and AMPK activity in TJ-41-treated myotubes, and did not find significantly elevated phosphorylation (Fig. 1C).

**Fig. 2:**
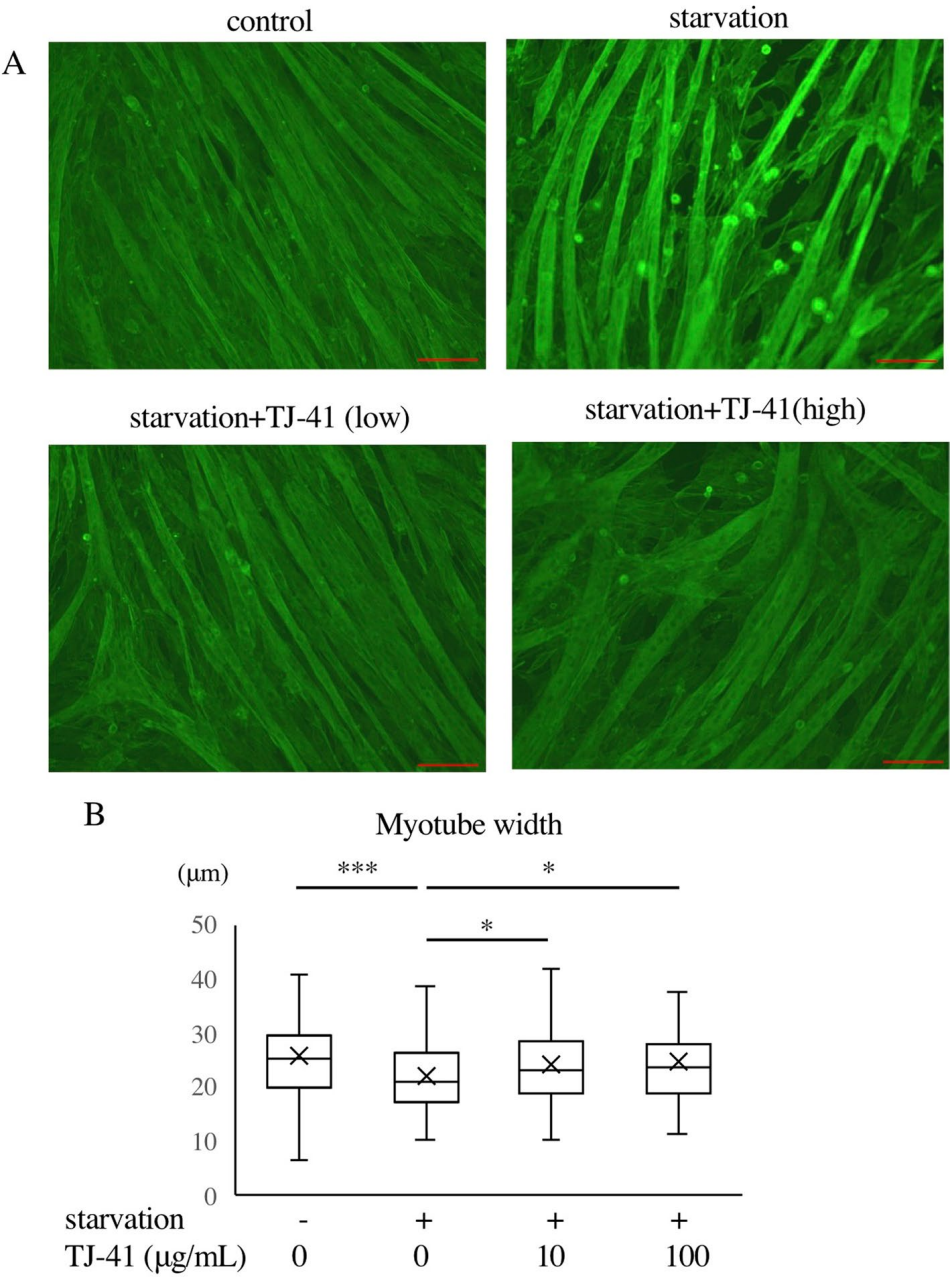
Effects of TJ-41 on C2C12 myotube morphology. **A** Representative images of C2C12 myotubes (20×). Differentiated C2C12 myotubes were subjected to serum starvation for 48 h with vehicle (DMSO) or TJ-41 extract (10 or 100 µg/mL). For visualization of cytoskeleton (F-actin), cells were stained with fluorescently-labeled phalloidin. Red bar indicates 100 μm. **B** Box plot of C2C12 myotube width in each group. The number of fibers analyzed in each group is 174, 158, 168, and 106, respectively. Significant differences were detected by the post-hoc Tukey-Kramer test. *p < 0.05, ***p < 0.001

Subsequently, we examined the effects of TJ-41 on C2C12 morphology. Following the method adopted in previous studies [32–34], multiple myotubes from each experiment were counted as samples, then their width was measured and statistically compared. The deprivation of serum for 48 h resulted in a decrease in myotube width, whereas TJ-41 treatment significantly inhibited this decrease in width (Fig. 2A, B).

### Effects of TJ-41 on muscles of tail-suspended mice

**Fig. 3:**
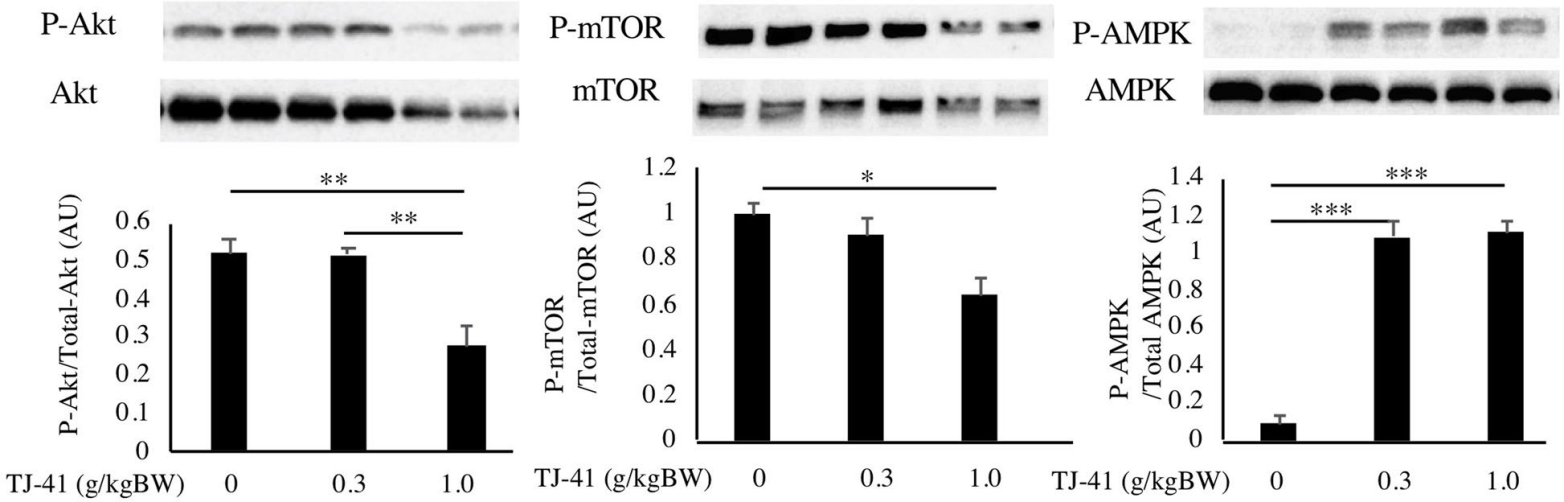
Effects of TJ-41 and tail-suspension on the gastrocnemius muscles. Phosphorylation of Akt, mTOR and AMPK in gastrocnemius muscles collected from control and TJ-41-administered mice. Mice were administered water (vehicle), a low-dose of TJ-41 (0.3 g/kgBW), or a high-dose of TJ-41 (1.0 g/kgBW) by gavage daily for 21 days (*n* = 4 in each group, total number is 12). Two samples from each treatment group were run and presented. These images were cropped from the original gels and blots. *p < 0.05, **p < 0.01

We administered TJ-41 (0.3 g/kgBW and 1.0 g/kgBW) to male wild-type mice for 21 days, followed by the isolation and analyses of the gastrocnemius muscles. Western blot analysis was performed using the muscle tissue samples to examine Akt, mTOR, and AMPK activity, respectively. ANOVA and post-hoc analysis showed that both treatment with 0.3 g/kgBW and 1.0 g/kgBW of TJ-41 significantly induced phosphorylation of AMPK (Fig. 3A). Whereas treatment with 0.3 g/kgBW of TJ-41 did not affect phosphorylation of Akt and mTOR, treatment with 1.0 g/kgBW of TJ-41 inhibited their phosphorylation.

Next, we subjected mice to tail-suspension and examined the atrogenes expression and changes in the weight of the gastrocnemius muscles. According to previous reports, MuRF1 and atrogin-1 levels peaked one day after the start of hindlimb suspension and gradually decreased thereafter, while muscle weight decreased monotonically until day 14 [35, 36]. Therefore, we measured MuRF1 and atrogin-1 expression of the gastrocnemius muscles after 24 h of hindlimb suspension and the muscle weight after 14 days of hindlimb suspension.

**Fig. 4:**
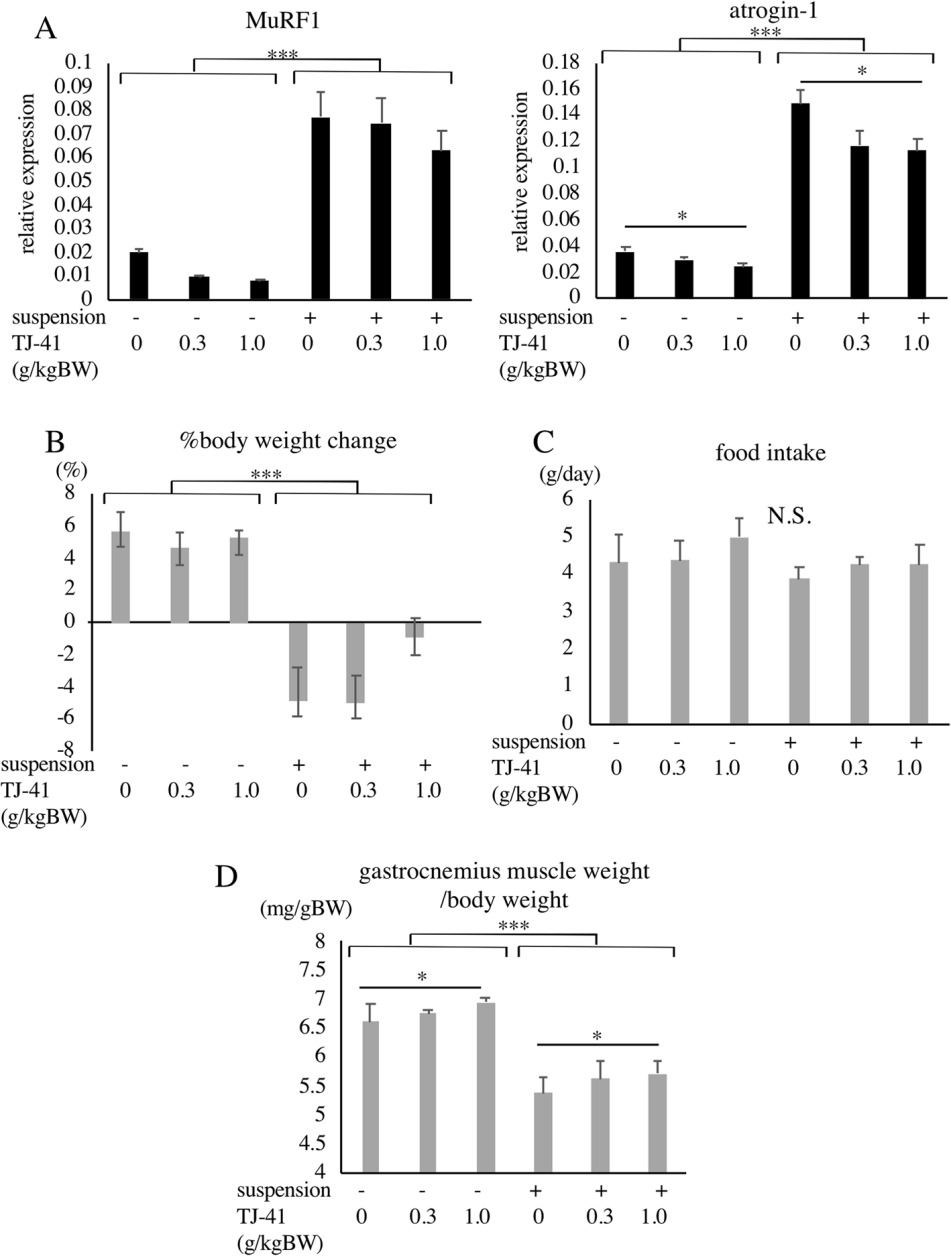
Effects of TJ-41 on tail-suspended mice. **A** Mice were subjected to 7 days of pretreatment and 24 h of tail-suspension. Effect of TJ-41 treatment on the expression of atrogin-1 and MuRF1 in gastrocnemius muscles of tail-suspended mice (*n* value is 10, 10, 10, 9, 10, 9 in each group, respectively, and total number is 58). **B**–**D** Mice were subjected to pretreatment for 7 days and tail-suspension for 14 days. The ratios of body weight changes during experiments (**B**), average food intake per day (**C**), and effects of TJ-41 treatment on the weight of gastrocnemius muscles (**D**). The low-dose and high-dose groups received 0.3 and 1.0 g TJ-41 per kg body weight, respectively (*n* value is 9, 10, 10, 9, 10, 8 in each group, respectively, and total number is 56). *p < 0.05, **p < 0.01, ***p < 0.001. NS indicates “not significant.”

In order to examine changes in atrogenes expression in response to TJ-41 in vivo, the mice were subjected to 7 days of pretreatment with TJ-41, followed by 24 h of tail-suspension. We performed a two-way ANOVA to see whether there is an interaction effect between tail-suspension and administration of TJ-41. Tail-suspension significantly increased MuRF1 expression in gastrocnemius muscles, but TJ-41 did not affect its expression (*p* = 0.158), and there were no interaction effects between them (*p* = 0.760) (Fig. 4A). Tail-suspension significantly increased atrogin-1 expression, TJ-41 significantly affected its expression, and there were no interaction effects between them (*p* = 0.111). Post-hoc analysis revealed that treatment with 0.3 g/kgBW did not downregulate atrogin-1, whereas treatment with 1.0 g/kgBW of TJ-41 significantly downregulated atrogin-1 expression.

Other groups of mice were subjected to 7 days of pretreatment with TJ-41, followed by 14 days of tail-suspension. We analyzed the effects of tail-suspension and TJ-41 on their body weight changes, food intake, and gastrocnemius muscle weight using two-way ANOVA. Although tail-suspension significantly decreased body weight, treatment with TJ-41 did not affect the body weights of the mice (Fig. 4B). The average food intake (per day) was not affected by tail-suspension and administration of TJ-41 (Fig. 4C). Intriguingly, treatment with 1.0 g/kgBW of TJ-41 significantly increased the weight of the gastrocnemius muscle in non-suspended mice and also significantly ameliorated weight loss in the muscles of tail-suspended mice (Fig. 4D). In all these experiments, no significant interaction between tail-suspension and administration of TJ-41 was detected.

## Discussion

In this study, we found that TJ-41 downregulated atrogin-1 expression both in vivo and in vitro, further revealing that TJ-41 activated AMPK in skeletal muscles, increased muscle mass in non-suspended mice and also ameliorated disuse-induced muscle atrophy in tail-suspended mice.

The high dose of TJ-41 used in the animal experiment in the present study, 1.0 g/kgBW, is equivalent to 4.6 g for a 60 kg person [37]. This amount is close to 5-7.5 g per day used in clinical medicine. Kampo formulas are usually given three times a day, but we administered TJ-41 once a day expecting that its blood levels would rise enough to be effective. However, there are no references about the dynamics of TJ-41 so far. Different administration methods could result in the difference of effects and this could be a limitation of the present study.

A previous study showed that TJ-41 ameliorated the loss of weight, cross sectional area (CSA) and isometric twitch force of gastrocnemius muscles in tail-suspended mice [22], which is compatible with the results of the present study. In the study, TJ-41 induced muscle differentiation and metabolism, upregulating myogenin and myosin heavy chain by downregulating NCoR1 expression in vitro and in vivo. We did not examine the effects of TJ-41 on NCoR1, but the novelty of our study is the suppressive effects of TJ-41 on atrogin-1. No association between NCoR1 and atrogenes has been reported. These multifaceted effects of TJ-41 may have resulted in increased muscle weight and ameliorated muscle atrophy.

Inhibition of atrogin-1 by TJ-41 was observed both in vivo and in vitro (Figs. 1B and 4A). While AMPK was activated and mTOR was inactivated in vivo, they were not affected in vitro (Figs. 1C and 3A). The results suggested that certain TJ-41 components have anti-atrophic effects including atrogin-1 suppression and amelioration of myotube width loss, independently of the PI3K/Akt/mTOR signaling pathway in vitro. On the other hand, it is known that many compounds present in the raw herbs are metabolized and converted by the host xenobiotic system and gut microbial flora [38], so it is suggested that they might be affected differently under in vivo and in vitro conditions, respectively, and the net effects of TJ-41 might be multifaceted.

Another novelty of our study is that TJ-41 activated AMPK in vivo. AMPK-mediated autophagy is known to be essential for maintaining muscle integrity and mitochondrial function during aging [39], and AMPK activation by exercise increased the expression of PGC-1α, and improved mitochondrial biogenesis and dysfunction, thereby restoring muscle function and inhibiting the loss of muscle mass [40]. In relation to herbal medicines, gosha-jinki-gan activated AMPK and induced its downstream PGC-1α expression, increasing the CSA of soleus muscle fibers in senescence-accelerated mice [11]. Ginseng pharmacopuncture extracts, which contain one of TJ-41 components ginseng, activated AMPK and induced differentiation markers such as MyoD and myogenin in vitro [41]. Thus AMPK activation by TJ-41 might be involved in the increase in the muscle mass.

Muscle weight increased in non-suspended mice administered TJ-41 for three weeks (Fig. 4D), whereas Akt and mTOR activity was decreased (Fig. 3A). The PI3K/Akt/mTOR pathway that promotes protein synthesis is supposed to be crucial in muscle hypertrophy [42]. AMPK is known to inhibit mTOR activation [7, 43]. However, both Akt and mTOR were inhibited, suggesting the pathway was suppressed upstream of Akt. Under overnutrient conditions, p70 inhibits insulin receptor substrate-1 (IRS-1) by phosphorylation at multiple sites, negatively regulating insulin signaling [44, 45]. This negative feedback might have inactivated Akt and mTOR on the day the mice were sacrificed.

In parallel, inflammation has also been suggested to be involved in atrogenes regulation, and serum TNF-α and IL-6 levels are increased in tail-suspended mice. [46, 47]. Activation of p38 and NF-κB by IL-1β induces expression of atrogin-1, independent of the Akt/FoxO pathway [48]. TJ-41 is suggested to reduce oxidative stress and inflammation in vivo [18, 23, 49]. Taken together, it is suggested that the anti-inflammatory effects of TJ-41 might be involved in the repression of atrogin-1. It might also be possible that the combination of this effect and NCoR1 suppression outweigh the effect of reduced mTOR activity, causing the increase in muscle weight.

Atrogin-1 is known to result in cachexia-induced muscle atrophy, and TJ-41 treatment ameliorates muscle weight loss in mouse cancer models together with decreased IL-6 production in macrophages [24], further suggesting that TJ-41 treatment might repress atrogin-1 in the cachexia-induced muscle atrophy.

## Conclusion

We found that TJ-41 treatment repressed atrogin-1 expression, thereby ameliorating disuse-induced muscle atrophy. TJ-41 might be a potential therapeutic agent against disuse-induced muscle atrophy.

## Supplementary Information

**Additional file** **1.**

## Data Availability

All data are included and described in this article. The datasets are not publicly available but are available from the corresponding author on reasonable request.

## Abbreviations

MuRF1: Muscle Ring finger 1
mTOR: Mammalian target of rapamycin
AMPK: 5’ adenosine monophosphate-activated protein kinase
PI3K: Phosphoinositide 3-kinase
FoxO: Forkhead box protein O
ATP: Adenosine triphosphate
DMEM: Dulbecco’s modified essential medium
FBS: Fetal bovine serum
DMSO: Dimethyl sulfoxide
RNA: Ribonucleic acid
cDNA: Complementary deoxyribonucleic acid
PCR: Polymerase chain reaction
GAPDH: Glyceraldehyde-3-phosphate dehydrogenase
SDS-PAGE: Sodium dodecyl sulfate-polyacrylamide gel electrophoresis
PBS: Phosphate buffered saline
TNF-α: Tumor Necrosis Factor-α
IL: Interleukin
NF-κB: Nuclear factor-κB
