# Supplementary material for: Kampo formula hochu-ekki-to (Bu-Zhong-Yi-Qi-Tang, TJ-41) ameliorates muscle atrophy by modulating atrogenes and AMPK in vivo and in vitro
Source: BMC Complement Med Ther. 2022 Dec 28;22:341. doi: 10.1186/s12906-022-03812-w (PMC9795672; doi:10.1186/s12906-022-03812-w)
Supplement: Supplementary file 1 — Additional file 1. [file 12906_2022_3812_MOESM1_ESM.docx]

The original, uncropped Western blots images

**Fig. 1C**

P-Akt


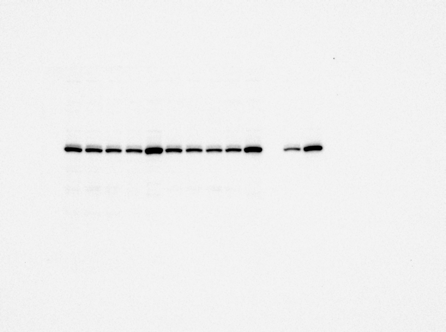


Akt


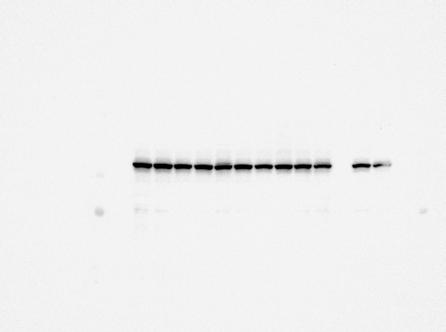


P-p70


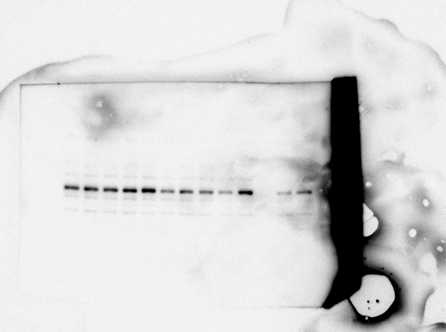


p70


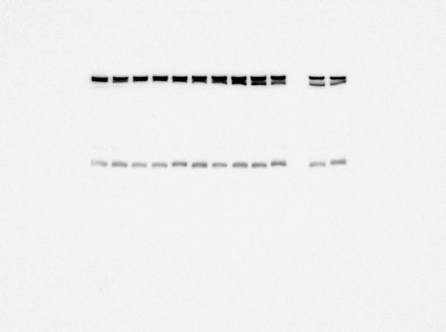


P-mTOR


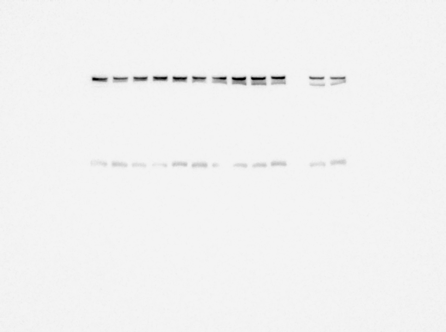


mTOR


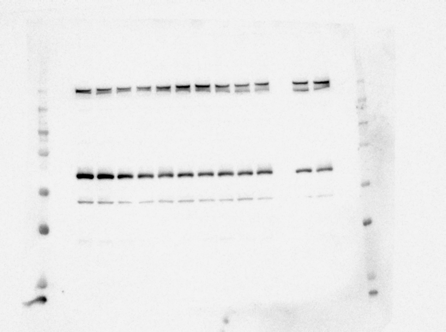


P-AMPK


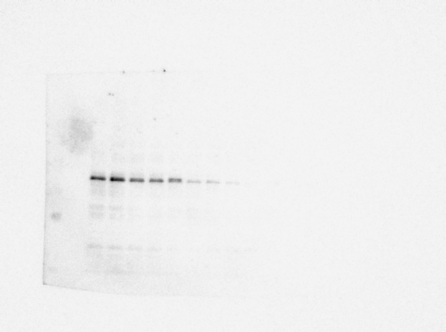


AMPK


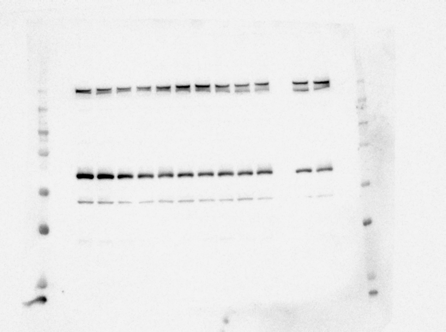


P-FoxO1


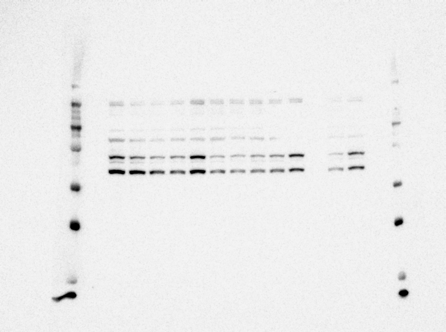


β-actin


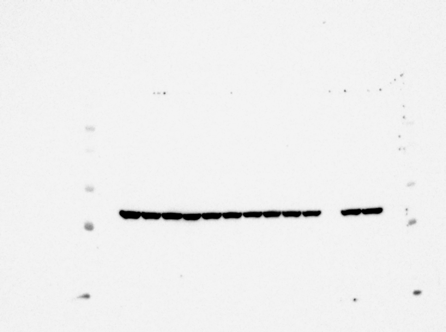


Western blots of the control and TJ-41-treated C2C12 myotubes are shown.

**Fig. 3**

P-Akt


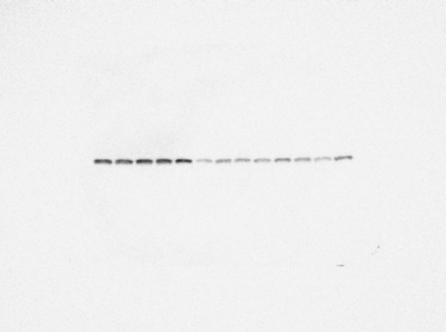


Akt


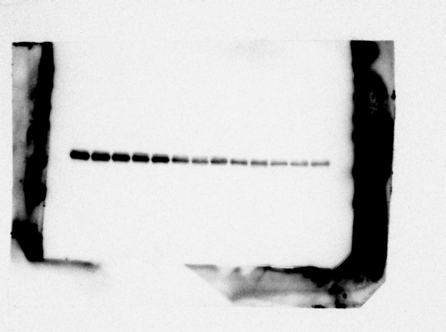


P-mTOR


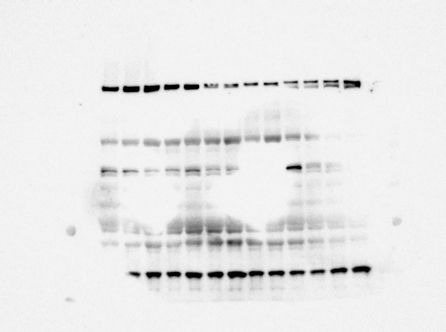


mTOR


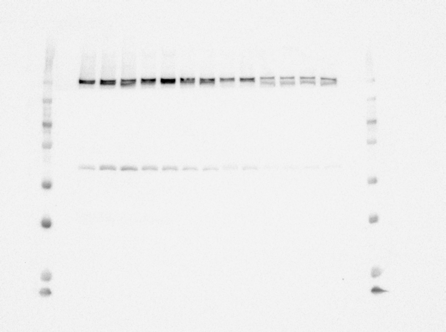


P-AMPK


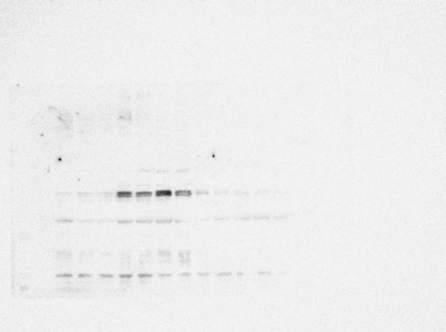


AMPK


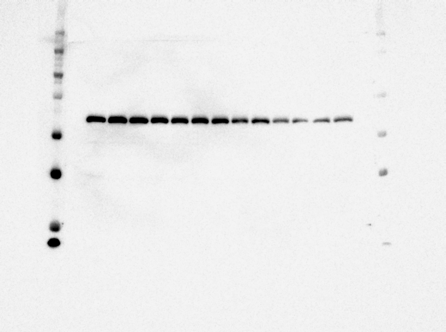


Akt, mTOR and AMPK (phosphorylated and total) in the gastrocnemius muscles collected from control and TJ-41-administered mice are shown.
